# Fabrication of a Floatable Micron-Sized Enzyme Device Using Diatom Frustules

**DOI:** 10.1021/acsomega.3c02104

**Published:** 2023-05-27

**Authors:** Nay San Lin, Kota Hirayama, Masaki Kitamura, Shinji Koide, Hiromasa Kitajima, Takunori Harada, Shigeki Mayama, Kazuo Umemura

**Affiliations:** † Department of Physics, Tokyo University of Science, 1-3 Kagurazaka, Shinjuku, Tokyo 1628601, Japan; ‡ Department of Integrated Science and Technology, Faculty of Science and Technology, Oita University, Dannoharu, 700, Oita City 870-1192, Japan; § Tokyo Diatomology Lab, 2-3-2 Nukuikitamachi, Koganei, Tokyo 184-0015, Japan

## Abstract

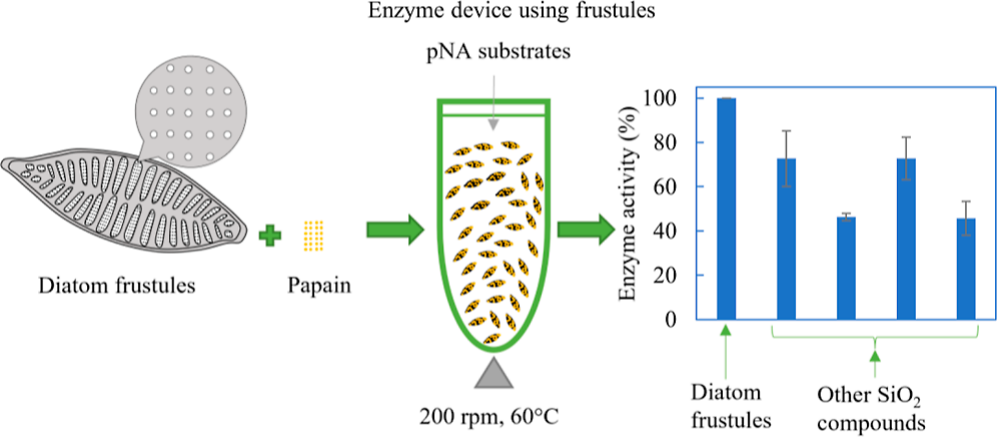

Immobilization of enzymes has been widely reported due to their reusability, thermal stability, better storage abilities, and so on. However, there are still problems that immobilized enzymes do not have free movements to react to substrates during enzyme reactions and their enzyme activity becomes weak. Moreover, when only the porosity of support materials is focused, some problems such as enzyme distortion can negatively affect the enzyme activity. Being a solution to these problems, a new function “floatability” of enzyme devices has been discussed. A “floatable” micron-sized enzyme device was fabricated to enhance the free movements of immobilized enzymes. Diatom frustules, natural nanoporous biosilica, were used to attach papain enzyme molecules. The floatability of the frustules, evaluated by macroscopic and microscopic methods, was significantly better than that of four other SiO_2_ materials, such as diatomaceous earth (DE), which have been widely used to fabricate micron-sized enzyme devices. The frustules were fully suspended at 30 °C for 1 h without stirring, although they settled at room temperature. When enzyme assays were performed at room temperature, 37, and 60 °C with or without external stirring, the proposed frustule device showed the highest enzyme activity under all conditions among papain devices similarly prepared using other SiO_2_ materials. It was confirmed by the free papain experiments that the frustule device was active enough for enzyme reactions. Our data indicated that the high floatability of the reusable frustule device, and its large surface area, is effective in maximizing enzyme activity due to the high probability to react to substrates.

## Introduction

Fabrication of micron-sized enzyme devices is one of the most advantageous and prevalently employed techniques for maximizing enzyme efficacy. In general, nanosized enzyme molecules are attached to the surface of micron-sized beads via chemisorption or physisorption.^1−4^

Micron-sized devices have at least two significant merits.^5,6^ The first is their reusability. Although enzyme molecules exhibit appropriate enzyme activity even when only dissolved in buffer solutions, it is not easy to reuse them. After an enzymatic reaction, product molecules, remaining substrate molecules, and enzyme molecules coexist in the solution. Because all the molecules are dissolved in the same solution, specific procedures, such as column chromatography, are required to recover the enzyme molecules.^7−9^ Although enzyme molecules can be reused, it is inconvenient to reuse them. If the enzyme molecules are attached to micron-sized materials, however, the micron-sized devices can be settled simply by using low-speed centrifugation. Because the substrate and product molecules are in the supernatant, settled enzyme devices can be easily recovered for reuse.

Second, several authors have described that the stability of enzyme molecules can be increased by integration into micron-sized devices. For example, Yang et al. succeeded in improving enzyme stability by immobilizing papain to micron-sized nanoporous silica.^10^ They suggested that papain molecules become embedded in the nanopores, stabilizing the papain structure. This was effective for the enzyme reuses.

The use of porous materials is another prevalent approach.^11−17^ Porous materials have larger surface areas than spheres or rods. The number of attached enzyme molecules can be dramatically increased because of the large surface area of porous materials. For example, Kuo et al. reported up to 95% loading of *Streptomyces griseus* HUT 6037 enzyme molecules.^11^ However, to our knowledge, there are no reports on the fabrication of enzyme devices using diatom frustules made of natural nanoporous biosilica, although there have been many reports of microbiodevices based on DEs and fossils of diatoms.^18−20^

Even though enzyme immobilizations on microbeads or porous materials have various merits, they still have problems to optimize. One of the problems of enzyme immobilization is that lower enzyme activity can be achieved compared to the native enzyme.^21^ Eş et al. pointed out that the immobilized enzymes have limited movements to react to the substrates due to the physical or chemical interactions with the support materials, unlike the free enzymes.^22^ Since the floatability of frustules is high even without external stirring, it was speculated that the enzyme activity of the enzyme device using frustules would be higher since immobilized enzymes on frustules would react more to substrates, compared to those immobilized on other support materials. Several authors have reported that porous support materials can enhance enzyme activity; however, only focusing on the porosity of support materials also has some disadvantages. One of those disadvantages is enzyme distortion, which can negatively affect the enzyme activity.^23^

Hence, in this study, to maximize the enzyme activity, we demonstrated the fabrication of micron-sized enzyme devices using frustules to add a new function, higher “floatability”, which means that the immobilized enzymes have better mobility to react to the substrates. Frustules are shells of diatom cells.^24^ Diatoms are major photosynthetic plankton found in seas, rivers, lakes, and other types of water bodies.^25^ It is known that diatoms produce 25% of the oxygen on Earth and approximately 19 billion tons of organic products.^26,27^ This means that large amounts of frustules can be obtained from nature, while isolated diatom cells can be cultured in laboratories.^28^ The specific gravity of living diatom cells is typically 1.3.^28,29^ This is much lower than that of typical micron-sized materials such as glass beads (2.5–5.5).^30,31^ Thus, although diatom cells will settle if there is no perturbation, they are floatable in the case of low perturbation. In enzyme devices using microbeads, sufficient stirring is necessary during enzyme reactions. Without stirring, the beads easily settle, and enzyme activity also decreases. Stirring with such magnetic stirrers is effective, but uniform stirring at the corners of a cuvette and the upper area of the cuvette is hard to realize. In some instruments, either stirring or heating should be selected. If micron-sized devices have higher floatability, it is greatly advantageous. We believe that higher floatability can be achieved in micron-sized enzyme devices using frustules.

It is known that almost 100,000 species of diatoms exist on Earth.^32^ The sizes of diatoms typically vary from several microns to several hundred microns.^33^ Frustules are nanoporous biosilica; thus, there are many nanosized pores on their surfaces.^34−36^ Their nanoporous structure is one reason for the low specific gravity of diatom cells. Furthermore, the size and shape of frustules are precisely regulated by their genes. In the case of chemically synthesized materials, purification is necessary to obtain uniform sizes.

DE is also made of nanoporous silica; therefore, there are reports on the fabrication of micron-sized enzyme devices.^37−39^ This material is advantageous for nanoporous micron-sized silica. However, there are essential differences between DE and frustules. DE is from the fossilized remains of diatoms; therefore, it can occur as blocks or aggregates. In addition, various diatom species are present in many cases. Thus, the size and shape of DE are not generally uniform. Furthermore, the specific gravity of DE is not uniform because it includes blocks and aggregates. Although DE provides a large surface area, from the viewpoint of “floatability,” DE and frustules are completely different materials.

We employed papain as the model enzyme in this study. Papain is a major cysteine proteinase widely used for medical and food applications.^40,41^ Although we believe that our proposal is applicable to various enzymes and other functional molecules, we chose papain enzymes for a specific reason. The optimal temperature for many human enzymes is 37 °C. Papain produces good enzyme activity at 37 °C; however, it can also operate at temperatures above 60 °C.^42^ For this reason, the “floatability” of our micron devices can be evaluated even at 60 °C with sufficient enzyme activity if papain enzyme is employed.

## Results and Discussion

**Figure 1:**
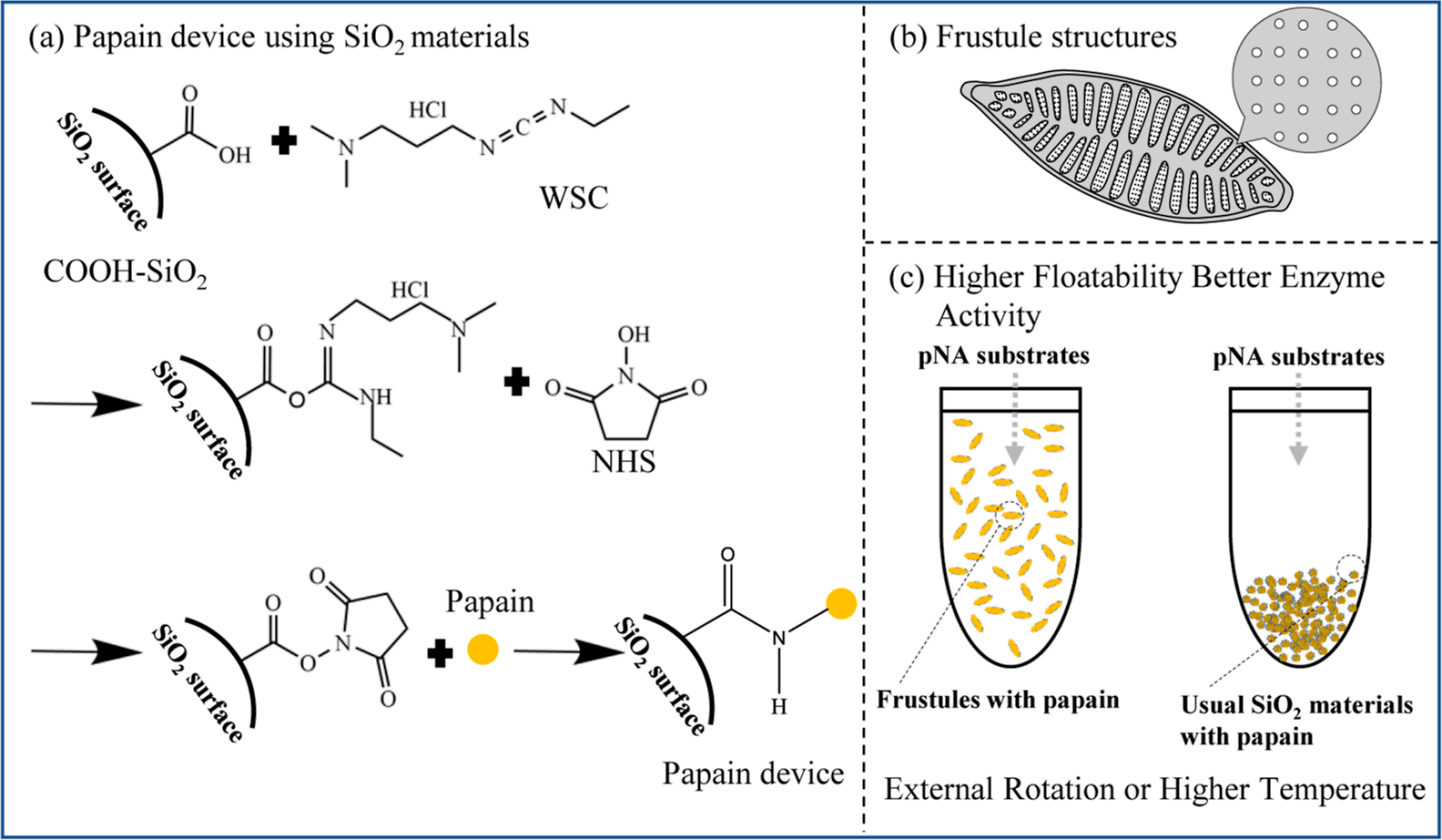
Schematic diagram of (a) floatable microbiodevices in which papain molecules are chemically crosslinked to SiO_2_ materials, (b) structure of frustules, and (c) overview of the research.

Figure 1 shows a schematic diagram of our experiments. Five types of SiO_2_ materials, frustules, mesoporous silica, DE 1, DE 2, and glass beads, were first functionalized with carboxyl-terminated silanes; then, papain molecules were attached to them using cross-linking agents (Figure 1a). Isolated diatom cells were cultured, and frustules were purified using a previously described procedure. Frustules have nanoporous structures and are several tens of microns in length (Figure 1b). The other four SiO_2_ materials were commercially available. We expected the frustule enzyme device to be more floatable compared to enzyme devices using the other four types of SiO_2_ materials. Therefore, we expected that the fabricated frustule device would have better mobility to react to the substrates, compared to other SiO_2_ devices, and hence, higher enzyme activity was expected for the frustule device. Enzyme reactions with the pNA substrates, named Bz-L-Arg-pNA·HCl [L-BAPA] substrates, were performed in sample tubes (Figure 1c), and enzymatic activity was evaluated by absorbance spectra. Conditions of external shaking and heating were varied to evaluate their effect on the floatability of the devices.

**Figure 2:**
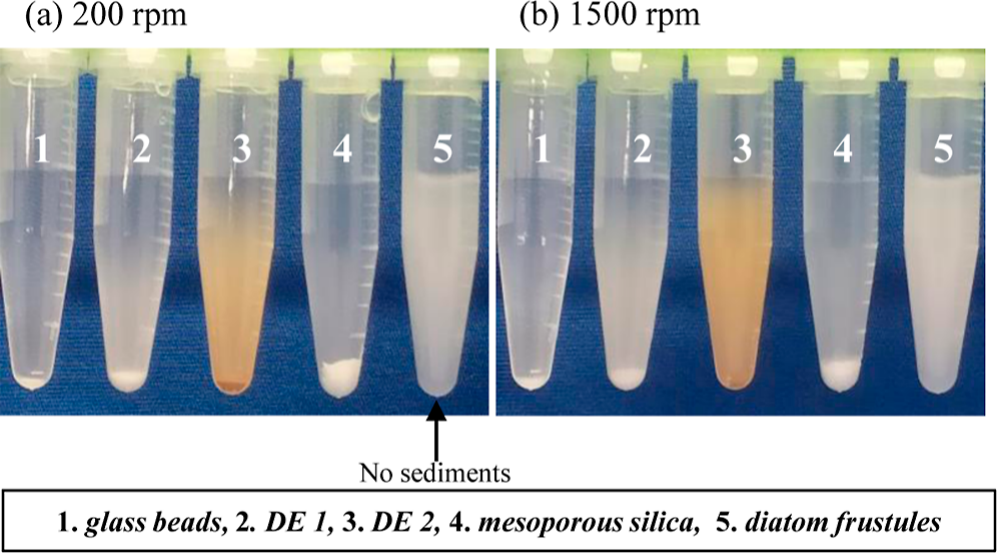
Floatability of SiO_2_ materials (5 mg/mL): glass beads, DE 1, DE 2, mesoporous silica, and diatom frustules (left to right) due to (a) 200 and (b) 1500 rpm at 25 °C.

The focus of this research was the fabrication of “floatable biodevices” using frustules. Figure 2 shows the floatability of the five types of SiO_2_ devices. Five milligrams of each SiO_2_ material were deposited in 1 mL of pure water and suspended by shaking with hand. The uniform suspension was immediately placed in a thermoshaker, and the samples were shaken at 0–1500 rpm for 2 min. In this experiment, bare SiO_2_ materials were used without papain attached.

Figure 2a,b shows photographs taken after 2 min of shaking at 200 and 1500 rpm, respectively. It is clear that frustules are present as a suspension, even at 200 rpm (see the arrow in Figure 2a). Pellets were observed for the other four types of SiO_2_ materials, even at 1500 rpm (Figure S1). The experiments were independently repeated three times. The frustules floated easily under all rpm conditions. In contrast, the glass beads did not form a suspension, even at 1400 rpm. The other three SiO_2_ materials gradually formed a suspension with increasing rpm; however, they were not perfectly suspended even at 1500 rpm. For example, in the case of DE 2, almost all the materials were well suspended, but some of those did not float. A series of photographs are shown in Figure S1. A photograph of a sample stored overnight without shaking is shown in Figure S2.

The morphology of each SiO_2_ material was examined using scanning electron microscopy (SEM, Figure S3). The frustules were approximately 29 μm in length and 6 μm in width (Figure S3a). Because frustules were purified by chemical treatments, the epitheca and hypotheca of frustules were basically separated from each other. A nanoporous structure can also be observed (Figure S3b). The diameter of the nanopores is 192.3 ± 40.6 nm (average of 50 pores). The diameter of the mesoporous silica is 437.9 ± 120.3 nm (average of 50 particles) (Figure S3c). The nanoporous structure of DE 1 and 2 is confirmed, but the sizes and morphology are not uniform because they are a mixture of various types of diatom fossils (Figure S3d–g). Some DEs are aggregated. The diameter of the glass beads is approximately 60 μm (Figure S3h,i).

**Figure 3:**
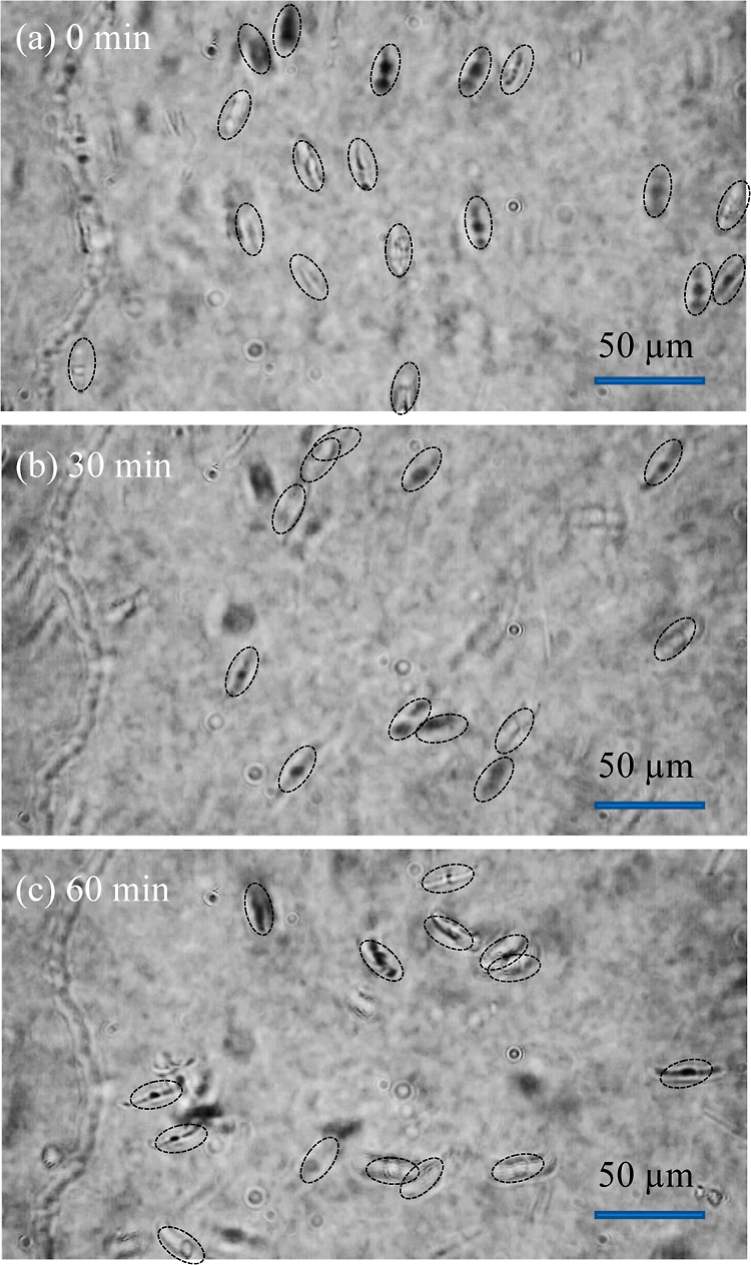
Observation of floatability of frustules at room temperature and at (a) 0, (b) 30, and (c) 60 min by the “tumbled” optical microscope.

Floatability of frustules was directly evaluated by a “tumbled” microscope in which a vertical sample stage can be viewed against the horizontal table.^28^ We set up a commercially available inverted microscope on a lab-made stand with 90° tilting. Using this microscope, floating/settling phenomena of micron-sized objects in liquids can be observed, in a way that fish can be observed in an aquarium. Figure 3 shows several captured images of frustules in water. When the frustule suspension was stored at room temperature for 1 h without any perturbation, many frustules remained floating in the water (Figure 3c), although the number of frustules decreased slightly. When similar experiments were performed at 30 °C, the frustules rapidly floated even after 1 h of observation. Movies of the floating frustules are shown in Movies S1 and S2. Although other materials were examined by a similar procedure, DE 1, DE 2, and glass beads were not observed to float because they rapidly settled. The mesoporous silica was too small to be observed using our microscope system.

For the preparation of enzyme devices, papain molecules were added to the SiO_2_ materials using the above-mentioned procedure (Figure 1). To verify the attachment of papain molecules, the absorbance of unbound papain molecules in the supernatant after centrifugation was measured (Figure S4). Nearly 90% of the papain molecules were added to each SiO_2_ surface in most cases. The results showed that when the amount of papain added was 0.08–1.12 mg per milligram of SiO_2_ materials, there were no significant differences between the five types of SiO_2_ materials. Thus, the number of added papain molecules to SiO_2_ surfaces was almost equal under these conditions. We used the condition of 0.53 mg of papain per milligram of SiO_2_ materials for the following enzyme assay experiments. Frustules decorated with papain were observed by atomic force microscopy (AFM) in air. Obscure spheres were observed on the frustule surfaces, and the bare frustule surfaces were rough (Figure S5).

The enzymatic activity of the fabricated micron-sized devices was evaluated by a hydrolysis assay using the pNA substrates. The papain devices were suspended in a (2-(*N*-morpholino)ethanesulfonic acid) (MES) buffer solution in a sample tube. The final concentrations of papain, SiO_2_ materials, and pNA were estimated to be 0.25 mg/mL (10.87 μM), 412.5, and 43.5 μg/mL (0.1 mM), respectively. Hence, the enzyme activity of the papain devices was measured with a much lower concentration of SiO_2_ materials compared to those in Figures 2 and S1. The samples were incubated at different rpm values and temperature conditions.

**Figure 4:**
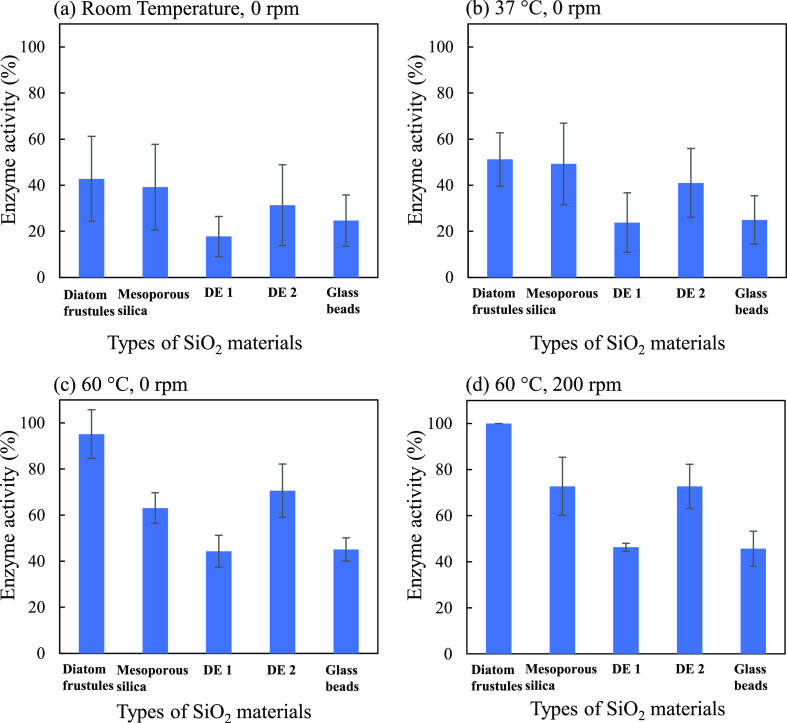
Enzyme activity of the fabricated SiO_2_ devices due to their floatability under four conditions: (a) room temperature, 0 rpm; (b) 37 °C, 0 rpm; (c) 60 °C, 0 rpm; and (d) 60 °C, 200 rpm. Error bars indicate standard errors.

Figure 4 shows histograms of enzyme activity for five independent experiments. If pNA is hydrolyzed by the papain enzyme, the absorbance at 405 nm should increase. When the sample was incubated at room temperature with no stirring (0 rpm), the enzyme activity was less than 50% in all the samples (Figure 4a). It was a too low temperature to obtain sufficient enzyme activity. When the incubation temperature was increased to 37 or 60 °C, the enzyme activity increased for all types of SiO_2_ materials (Figure 4b,c). When the samples were shaken at 200 rpm, it was also effective for improving the enzyme efficiency. In all the conditions, the frustule enzyme device exhibited the highest enzyme activity since it is speculated that the immobilized papain molecules on the frustules had fewer limitations in movements due to the high floatability of frustules, and hence, they could react better with the pNA substrates.

An analysis of enzyme activity is presented in Table S1. We defined the enzyme activity at 60 °C and 200 rpm as 100%. The activities of the frustule devices without shaking were 42.8, 51.4, and 95.0% at room temperature, 37, and 60 °C, respectively. As we described, the frustules floated very rapidly at 37 °C without stirring because of their low specific gravity. In addition, it is well known that papain exhibits good enzyme activity even above 60 °C because of its high thermostability. An increase in enzyme activity was observed with an increase in incubation temperature. Furthermore, an increase in enzyme activity was observed in the frustule devices in comparison to the other forms of SiO_2_ devices.

Compared to the enzyme activity of the frustule device at 60 °C and 200 rpm, the activities at room temperature with 0 rpm were 42.8, 39.2, 17.6, 31.1, and 24.8% in frustules, mesoporous silica, DE 1, DE 2, and glass beads, respectively. Similarly, the activities at 37 °C and 0 rpm were 51.4, 49.1, 23.9, 41.0, and 24.8% for frustules, mesoporous silica, DE 1, DE 2, and glass beads, respectively. Only DE 2 showed higher enzyme activity compared to that of the frustules. In an analysis of floatability, DE 2 exhibited better floatability than the other nonfrustule devices. The DE 2 device showed better enzyme activity than the other nonfrustule devices. The results also suggest that floatability is strongly related to the enzyme activity of the devices. The enzyme activity at 200 rpm and 60 °C was examined for comparison. There was no significant difference in the enzyme activity between 0 and 200 rpm at 60 °C.

**Figure 5:**
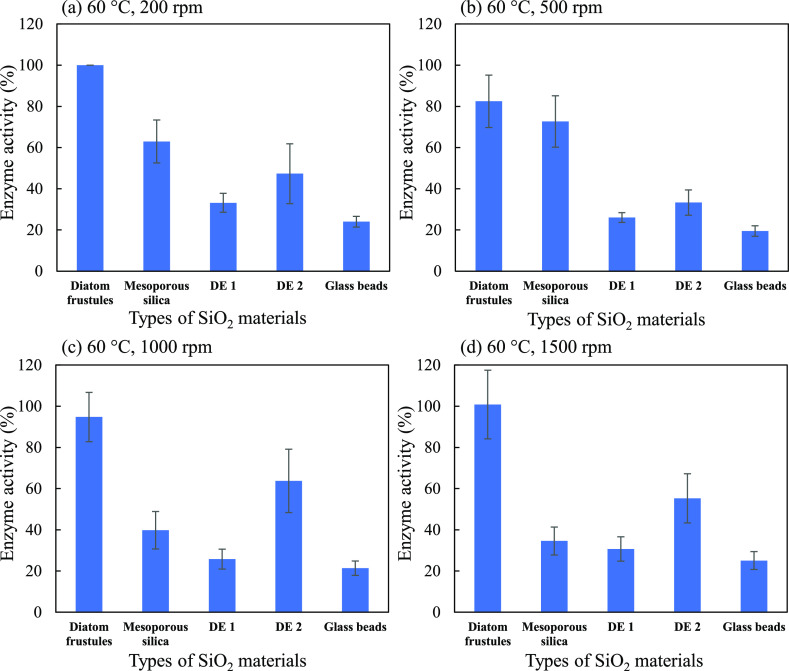
Enzyme activity of the fabricated SiO_2_ devices due to their floatability at (a) 60 °C, 200 rpm, (b) 60 °C, 500 rpm, (c) 60 °C, 1000 rpm, and (d) 60 °C, 1500 rpm. Error bars indicate standard errors.

We examined the effect of rpm on the enzyme activity of the SiO_2_ devices using the same method (Figure 5). However, the medium of performing the experiments may not be identical to the previous measurements. The frustule devices showed the highest enzyme activity in all rpm values and slow stirring speeds as 200 rpm might be enough to show the highest enzyme activities due to their high floatability even at low stirring speeds. At 500 rpm, a decrease in the enzyme activity of the frustules might be due to the loss of a slight number of frustules during the replacements of supernatants after centrifugation. SiO_2_ materials having low specific gravity, especially the frustules, have good floatability. Hence, the loss of a slight amount of them may occur during the replacement of supernatants. Only DE 2 devices showed higher enzyme activity at higher stirring speeds as 1000 and 1500 rpm. This result was consistent with the floatability analysis of the SiO_2_ materials (Figure S1). In Figure S1, aside from frustules which are well suspended even at 200 rpm, only DE 2 showed a very remarkable increase in floatability when the stirring speed was increased. The amount of SiO_2_ materials used might be one of the factors of no remarkable changes in the enzyme activity of other SiO_2_ materials despite the increased stirring speeds. In Figure S1, 5 mg/mL SiO_2_ materials were used to clearly illuminate their floatability. When the enzyme assays were performed, the added final concentration of SiO_2_ materials was only 412.5 μg/mL.

**Figure 6:**
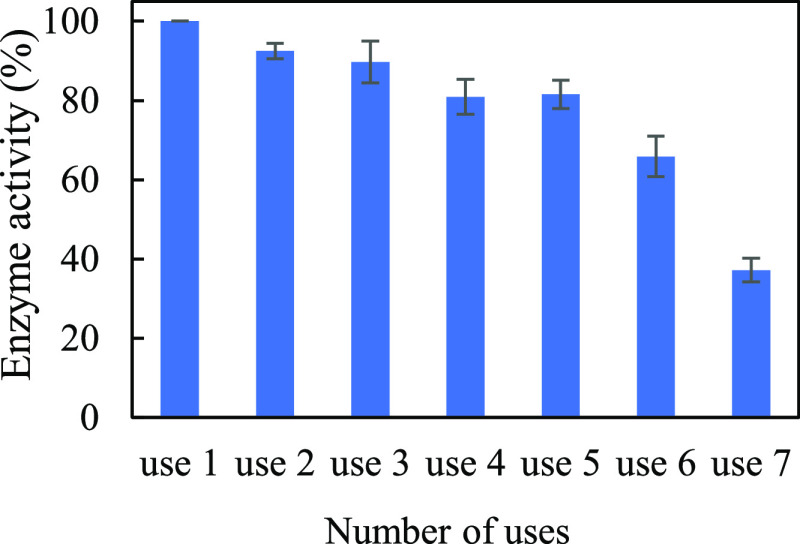
Reusability of immobilized papain on frustules. Error bars indicate standard errors.

Finally, the reusability of the frustule devices was evaluated at 60 °C and 200 rpm. In the experiments, the number of devices in the sample tube was increased to avoid variation in the data caused by adsorption of the devices to the walls of the tube. The final concentrations of papain, frustule, and pNA were 0.25 mg/mL, 1.24 mg/mL, and 43.5 μg/mL, respectively. The enzyme devices were recovered by centrifugation, and the enzymatic reaction was repeated seven times using the same sample. The reuse experiments were independently performed five times. Figure 6 shows the average enzyme activity. The results are listed in Table S4. If we assume that the enzyme activity of the first use was 100%, more than 80% of the activity remained after the fifth use.

We also analyzed the enzyme activity of free papain (Figure S6). Free papain was mixed with MES buffer (pH 7.0, 10 mM) in a microtube. Its enzyme activity was performed immediately without delay at 0 rpm and 60 °C. The added final concentration of the free papain was calculated as 0.25 mg/mL. Absorbances of the pNA substrates which reacted to the free papain were measured at 405 nm, which shows the activity of enzymes. The experiment was carried out five times. The average absorbance of the reacted pNA to the free papain was 0.156 ± 0.013.

Using the same papain used for the free papain experiments, we fabricated the enzyme device using frustules and analyzed its enzyme activity (Figure S7). However, papain was attached to the frustules under the MES buffer medium (pH 7.0, 10 mM) in a microtube. The suspension was incubated at room temperature using a rotator for 2 h. After that, the unattached papain was removed by centrifugation. Then, the enzyme activity of the frustule device was analyzed. Absorbances of the pNA substrates which reacted to the frustule device were measured at 405 nm five times. The average absorbance of the reacted pNA to the frustule device was 0.110 ± 0.012.

It was seen that the free papain showed its enzyme activity by focusing on the absorbance of reacted pNA substrates at 405 nm. Hence, it can be confirmed by the free papain experiments that the frustule device also showed good enzyme activity. However, direct comparisons between numerical values were avoided due to slightly different methods of performing the enzyme activity of the free papain and that of the fabricated enzyme device. Losito et al. also studied the absorbance of pNA substrates at 405 nm to confirm the activity of papain immobilized on mesoporous silica (SBA-15).^43^ Hence, we conclude that our frustule device was active enough for enzyme reactions.

In summary, we have developed a floatable enzyme device using frustules and natural nanoporous biosilica. Our method is likely to be applicable to various enzyme devices and other biological micron-sized devices. Furthermore, various micron-sized biodevices with different floating patterns could be fabricated using 10,000 diatom species.

## Conclusions

Even though enzyme immobilization on microbeads and porous support materials has various advantages, it also has problems improving. It has been reported that the enzyme activity of the immobilized enzymes was weak due to the limitation of their free mobility to react to substrates, as well as enzyme distortion in the pores. Hence, in this study, we fabricated a floatable papain device using natural nanoporous biosilica called frustules. The fabricated enzyme device floated perfectly at 30 °C, even without stirring. Based on a comparison with four other types of micron-sized SiO_2_-based materials, the high floatability of frustules is probably one of the important factors for maximizing enzyme activity. The papain device using diatom frustules exhibited the highest performance among the five candidates for micron-sized SiO_2_ materials. Hence, we have successfully reported an insightful application of nanoporous biosilica, frustules, in the fabrication of enzyme devices by highlighting its floatability’s influence on the enzyme activity.

## Materials and Methods

Five types of SiO_2_ materials were used: diatom frustules, mesoporous silica, DE 1 (white), DE 2 (red), and glass beads. Mesoporous silica (MCM-48, 805467-5G), DE 2 (D3877-500G), and trichloroacetic acid (TCA) solution (T0699-100ML) were purchased from Sigma-Aldrich Co. LLC (St. Louis, MO, USA). DE 1 (CAT no. 157606) was purchased from MP Biomedicals, LLC (France). Glass beads (GB-0.05) were purchased from Kenis, Ltd. (Osaka, Japan). Carboxyethylsilanetriol, disodium salt, 25% in water (COOH silane) (SIC2263.0-25GM) were obtained from Gelest Inc. (Morrisville, USA). 1-Ethyl-3-(3-dimethylaminopropyl)carbodiimide hydrochloride (WSC) (DOTITE 348-03631) and ethylenediamine-*N*,*N*,*N*′,*N*′-tetraacetic acid, trisodium salt trihydrate (3NA(EDTA·3H_2_O)) were purchased from Dojindo Laboratories (Kumamoto, Japan). *N*-Hydroxysuccinimide (NHS) (081-09771), papain powder (164-00172), l-cysteine hydrochloride monohydrate (033-05272), and 5 mol/L sodium hydroxide solution (NaOH) (196-05375) were purchased from Fujifilm Wako Pure Chemical Corporation (Osaka, Japan). Bz-L-Arg-pNA·HCl [L-BAPA] was purchased from Peptide Institute. Inc. (Osaka, Japan).

Diatom cells (*Craspedostauros* sp.) were collected from the seaside in Chiba Prefecture, Japan, and isolated. Guildard’s (f/2) marine water enrichment solution (G9903; Sigma-Aldrich, Munich, Germany) in Daigo artificial seawater (395-01343; Nihon Pharmaceutical, Tokyo, Japan) was used to subculture the diatom cells. The cultivation temperature ranged from 18 to 25 °C.^28,44^ Frustules (approximately 20–35 μm) from diatom cells were purified using nitric acid as follows: cell suspensions (9 L) were centrifuged five times at 1630*g* for 5 min each to remove the culture medium and decrease the volume. 100 mL of cell suspension was prepared in water. The volume of concentrated nitric acid was twice that of the cell suspension, and the samples were incubated for 40 min at 95 °C to degrade the organic components. A spoonful of a medical spoon of potassium nitrate was added to the cell suspension as soon as possible and diluted with ice water to stop the reaction. The suspension was centrifuged five times at 1630*g* for 5 min each to remove the nitric acid.

Five 1 mL SiO_2_ suspensions were prepared in microtubes (1.5 mL) at a concentration of 5 mg/mL. The suspensions were stored at room temperature (23 °C) overnight so that the SiO_2_ particles were precipitated at the bottom of the microtube. The suspensions were homogenized with an Iuchi automatic lab mixer (HM-10H, Japan) and shaken at 200–1500 rpm using a thermoshaker (MSC-100, Chiyoda Science Co., Ltd, Tokyo, Japan) at 25 °C. The shaking period was set to 2 min after homogenization using the lab mixer. The suspensions were photographed immediately using a camera.

The structural properties of the five types of SiO_2_ materials were examined by SEM (JSM-6510, JEOL Ltd., Tokyo). The samples were added dropwise onto coverslips and dried at room temperature for 1 h. The samples were sputtered using an MSP-1S device (Vacuum Device Inc., Ibaraki, Japan) with a gold–palladium (Au–Pd, 4:6) target. The sizes of the particles and pores were determined.

A homemade “tumbled” optical microscope was employed to observe the floatability of frustules at different temperatures. The purified frustules were adjusted to a concentration of 0.1 mg/mL with ultrapure water. A frustule suspension (1 mL) was placed in a cuvette and observed under a microscope (40× objective lens, HDR-CX590 video camera, SONY, Tokyo, Japan). Observations were performed for 1 h at room temperature and 30 °C using the same sample. Movies of the frustules floating at these temperatures were recorded.

Before papain attachment, 10 μL of COOH silane and 1 mg of SiO_2_ materials were mixed in 990 μL of pure water. The mixture was incubated at room temperature for 1 h using a rotator. The suspension was centrifuged at 5000*g* and 25 °C for 3 min and washed with 700 μL of MES buffer (10 mM, pH 7.0) six times. 300 μL of the silanized SiO_2_ suspension (COOH–SiO_2_) was stored.

COOH–SiO_2_ (250 μL) and MES buffer (250 μL, 10 mM, pH 7.0) were mixed in a microtube. Also, 25 mg of NHS and 15 mg of WSC were mixed with 250 μL of MES buffer (10 mM, pH 7.0). The two suspensions were mixed in a microtube such that the volume of the final functionalized SiO_2_ materials was 750 μL. The suspension was centrifuged at 5000*g* at 25 °C for 3 min and washed with 650 μL of MES buffer (10 mM, pH 7.0) six times. 100 μL of the functionalized SiO_2_ suspension (final concentration: 8.25 mg/mL) was stored.

Papain powder was dissolved in MES buffer, and the stock solution (10 mg/mL) was stored on ice. Five types of SiO_2_ materials (1 mg/mL) and five concentrations of papain solution (1, 3, 5, 7, and 10 mg/mL) were chosen for papain–SiO_2_ assays. The prepared functionalized SiO_2_ materials (50 μL), papain solution (50 μL), and MES buffer (900 μL) (10 mM, pH 7.0) were mixed in a microtube. The final concentrations of papain used were 0.05, 0.15, 0.25, 0.35, and 0.5 mg/mL. After incubating the mixtures at room temperature for 2 h by gentle rotating, the samples were centrifuged at 5000*g* at 25 °C for 3 min. To estimate the amount of unbound papain, after centrifugation for the first time, the absorbance of the first supernatant (750 μL) was measured using a Duetta spectrometer (Horiba Ltd., Kyoto, Japan) in the wavelength region of 250–600 nm because the absorption peaks of proteins are reportedly observed at approximately 280 nm.^45^ The experiments were performed in triplicate. The concentration of the unattached papain on SiO_2_ materials was determined by
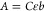
1where *A* is the absorbance of the supernatant, *C* is the concentration of the unattached papain, ε (57,628 M^–1^ cm^–1^)^46^ is the molar absorption coefficient of papain, and *b* is the thickness of the cuvette. Immobilization efficiency (IE) was calculated by
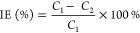
2

Adsorption capacity, *Q* (mg/mg), of papain on SiO_2_ materials was determined by
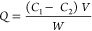
3where *C*_1_ and *C*_2_ are the total final concentration of papain used for enzyme reactions (mg/mL) and that of the unattached papain in the supernatant (mg/mL). *V* is the total volume of suspension (mL), and *W* is the final weight of SiO_2_ materials (mg) used for enzyme reactions.

Bare frustules and frustules functionalized with papain were evaluated using an atomic force microscope (MFP-3D microscope, Asylum Research, Santa Barbara, CA, USA). Fifty microliters of frustule suspensions, 50 μL of the papain solution (final concentration: 0.25 mg/mL), and 900 μL of MES buffer (pH 7.0, 10 mM) were placed in a microtube and shaken for 2 h. The suspension was washed six times with pure water at 5000*g* at 25 °C for 3 min. The same concentration of the frustule suspension (only frustules suspended in water) was used to analyze the structure of the bare frustules. The AC-AFM mode in air was chosen for the samples by applying a silicon cantilever PPP-NCSTR-W (Nanosensors, Nanoworld AG, Neuchatel, Switzerland) and mica substrates treated with 0.01% 3-aminopropyl triethoxysilane (Shin-Etsu Chemical Co., Ltd. Tokyo, Japan). The sample (10 μL) was added dropwise onto the mica substrate and dried using a dryer for 15 min.

Papain immobilization onto the functionalized SiO_2_ materials was carried out by the method described above but by using 50 μL of a 5 mg/mL papain solution (final concentration: 0.25 mg/mL). Unattached papain was removed by washing the suspension with 700 μL of MES buffer (10 mM, pH 7.0) six times at 5000*g* at 25 °C for 3 min, and 630 μL of MES buffer was replaced the last time instead of 700 μL so that the final volume of the papain devices would be 930 μL.

0.44 g of l-cysteine hydrochloride monohydrate powder, 0.27 g of EDTA trisodium salt, and 45 mL of pure water were homogenized using a magnetic stirrer. The pH level of the solution was adjusted to 7.5 by adding a sodium hydroxide (NaOH) solution dropwise while stirring with a magnetic stirrer. This activation solution was stored in a freezer (0 °C).

The papain devices (930 μL) were activated by injecting 50 μL of the cysteine solution (final concentration: 0.03 M). To focus on the effects of temperature and rpm on the floatability of SiO_2_ materials, the activated papain devices were incubated for 1 h at different temperatures (room temperature, 37 °C, and 60 °C) with no shaking (0 rpm) or 200 rpm external stirring using a thermoshaker.

After incubation for 1 h, 10 μL of the pNA substrate (final concentration: 0.1 mM) was injected into the activated papain devices and incubated for 1 h at the same temperature and shaking speed. 10 μL of TCA (final concentration: 0.05% m/v) was added to the tube to stop the enzyme reactions, and the sample was centrifuged once at 5000*g* and 25 °C for 3 min to collect the supernatant. The absorbance of the supernatant was measured by the Duetta spectrometer (Horiba, Ltd., Kyoto, Japan) in the 250–600 nm wavelength range. The absorbance of the pNA substrates reacted with papain immobilized on the SiO_2_ materials at 405 nm was focused.^47^ The experiment was repeated five times.

Stirring effects on the enzyme reactions were evaluated again using the fabricated SiO_2_ devices at 60 °C and 200, 500, 1000, and 1500 rpm. The experiments were carried out using the same method but not in an identical medium in which room temperature, humidity, and so forth may be slightly different from those of the above experiments.

Papain molecules were attached to 3 mg/mL of frustules by chemisorption using the method described above, and the enzyme reactions were performed and analyzed using the Duetta spectrometer. Then, the enzyme devices were washed three times with 10 mM MES buffer (pH 7.0) at 5000*g* at 25 °C for 3 min for reuse. The same methodology was used to examine the activity of the enzyme devices up to seven reuses for each sample. The experiments were repeated five times for each sample. The enzyme reactions of each reuse experiment relative to the first were compared.

For the enzyme activity of free papain, a 5 mg/mL papain solution was prepared. 50 μL of the 5 mg/mL papain solution and 880 μL of MES buffer (pH 7.0, 10 mM) were mixed in a microtube. After adding 50 μL of a cysteine solution, the enzyme activity was performed immediately without delay at 60 °C and 0 rpm. Further procedures of enzyme activity were performed the same as mentioned above. A UV–vis spectrophotometer (V-630, JASCO Corp., Hachioji City, Tokyo, Japan) was exploited in the wavelength region of 250–600 nm. Absorbances of reacted pNA substrates to the free papain were focused on 405 nm. The experiments were carried out five times.

Using the same papain utilized for the free papain experiments, the enzyme devices were fabricated on the same day. The above-mentioned procedures of fabricating the frustule device were followed; however, the medium of conducting experiments might not be identical. A UV–vis spectrophotometer (V-630, JASCO Corp., Hachioji City, Tokyo, Japan) was exploited in the wavelength region of 250–600 nm to measure the absorbance at 405 nm. The experiments were carried out five times.

## Supporting Information Available

Evaluation of floatability of SiO_2_ materials; photographs of fully settled SiO_2_ materials; SEM of SiO_2_ materials; attachment of papain molecules to functionalized SiO_2_ materials; AFM images of frustules with and without papain; enzyme activity of free papain; enzyme activity of the frustule device; tables of enzyme activity of the immobilized papain at different temperatures; adsorption capacity of papain on SiO_2_ materials; enzyme activity of the immobilized papain at different stirring speeds; and reusability of the enzyme devices using frustules (PDF)
Direct observation of floating frustules (MP4)

## Abbreviations

3NA(EDTA·3H_2_O): ethylenediamine-*N*,*N*,*N*′,*N*′-tetraacetic acid, trisodium salt trihydrate
AFM: atomic force microscopy
COOH: carboxyethylsilanetriol, disodium salt, 25% in water
DE: diatomaceous earth
MES: 2-(*N*-morpholino)ethanesulfonic acid
NHS: *N*-hydroxysuccinimide
pNA: *p*-nitroaniline, Bz-L-Arg-pNA·HCl [L-BAPA]
SEM: scanning electron microscopy
SiO_2_: silicon dioxide
TCA: trichloroacetic acid
WSC: 1-ethyl-3-(3-dimethylaminopropyl)carbodiimide hydrochloride
