# Correction to “Fabrication of a Floatable Micron-Sized Enzyme Device Using Diatom Frustules”

**DOI:** 10.1021/acsomega.3c09272

**Published:** 2023-11-30

**Authors:** Nay San Lin, Kota Hirayama, Masaki Kitamura, Shinji Koide, Hiromasa Kitajima, Takunori Harada, Shigeki Mayama, Kazuo Umemura

In the original article, in the third sentence of the sixth paragraph in the “MATERIALS AND METHODS” section, “700 μL of MES buffer (10 mM, pH 7.0)” was written instead of “700 μL of pure water” (Line 9, Column 1, Page 21151). This small mistake due to oversight has been rectified below. The authors apologize for the error.

Rectified Sentence in the Sixth Paragraph in “MATERIALS AND METHODS” Section:

The suspension was centrifuged at 5000*g* and 25 °C for 3 min and washed with 700 μL of pure water six times.

