# Supplementary material for: Fabrication of a Floatable Micron-Sized Enzyme Device Using Diatom Frustules
Source: ACS Omega. 2023 May 27;8(23):21145–53. doi: 10.1021/acsomega.3c02104 (PMC10268610; doi:10.1021/acsomega.3c02104)
Supplement: Supplementary file 1 — ao3c02104_si_001.pdf [file ao3c02104_si_001.pdf]

Supporting Information

Fabrication of a Floatable Micron-sized Enzyme Device Using Diatom Frustules

Nay San Lin[1], Kota Hirayama[1], Masaki Kitamura[1], Shinji Koide[1], Hiromasa Kitajima[2], Takunori Harada[2], Shigeki Mayama[3], Kazuo Umemura[1]*

[1]Department of Physics, Tokyo University of Science, 1-3 Kagurazaka, Shinjuku, Tokyo 1628601, Japan

[2]Department of Integrated Science and Technology, Faculty of Science and Technology, Oita University, Dannoharu, 700, Oita City 870-1192, Japan

[3]Tokyo Diatomology Lab, 2–3-2 Nukuikitamachi, Koganei, Tokyo 184-0015, Japan

*1221711@ed.tus.ac.jp

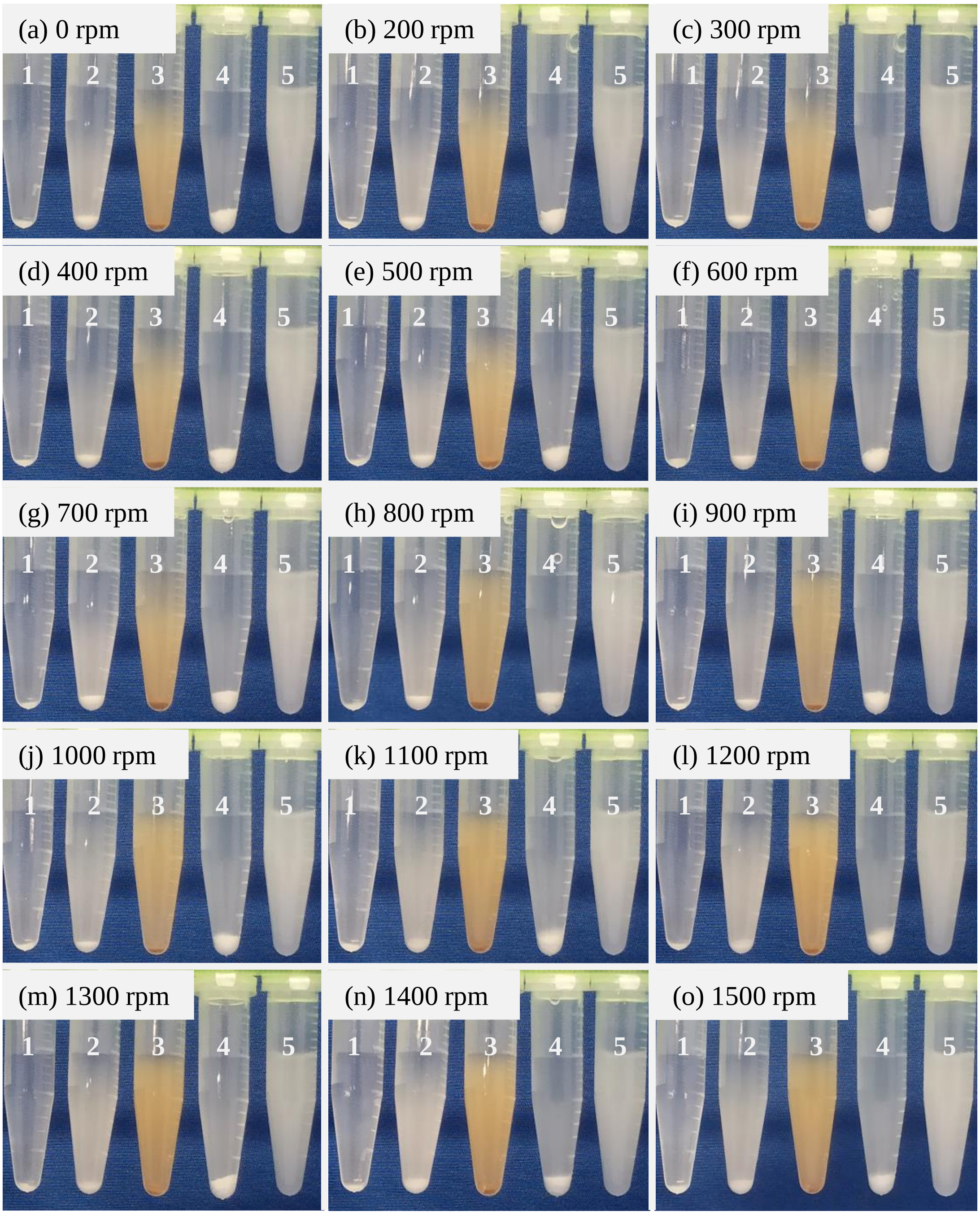

1. glass beads, 2. DE 1, 3. DE 2, 4. mesoporous silica, 5. diatom frustules

Figure S1

Evaluation of floatability of SiO_2 materials.

Floating abilities of five types of SiO_2 materials due to shaking speeds (rpm). From left to right, the types of SiO_2 materials are glass beads, DE 1, DE 2, mesoporous silica and diatom frustules. Figures (a-o) represent the SiO_2 suspensions after shaking at different shaking speeds (0-1500 rpm) for 2 min. Prior to this shaking at different rpm values, samples were fully suspended by hand shaking. Photos were taken as soon as the shaking was finished.

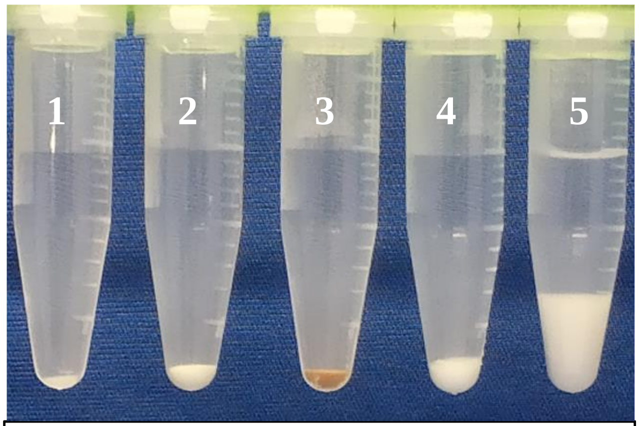

1. glass beads, 2. DE 1, 3. DE 2,
4. mesoporous silica, 5. diatom frustules

Figure S2

Photographs of fully settled SiO_2 materials.

SiO_2 material suspensions were stored overnight without stirring.

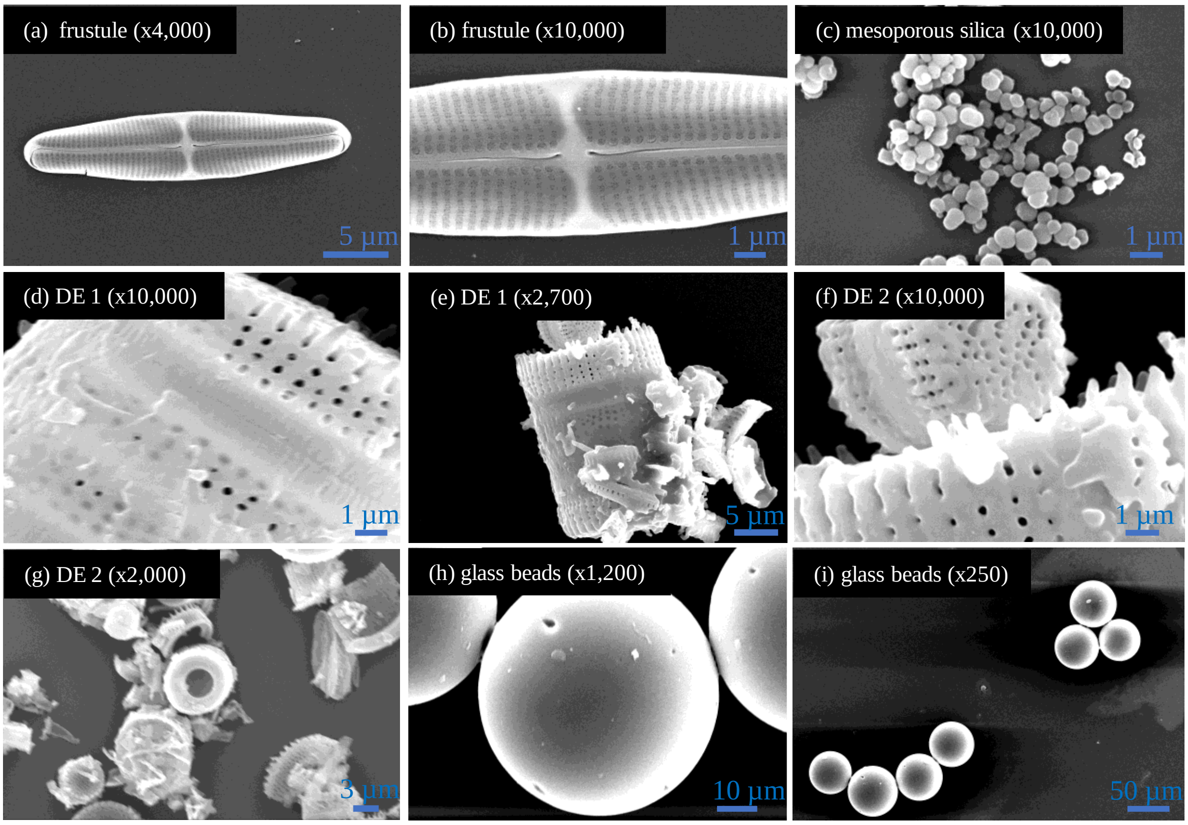

Figure S3

Scanning electron microscopy of SiO_2 compounds.

SEM images of (a) diatom frustule (×4,000), (b) diatom frustule (×10,000), (c) mesoporous silica (×10,000), (d) DE 1 (×10,000), (e) DE 1 (×2,700), (f) DE 2 (×10,000), (g) DE 2 (×2,000), (h) glass beads (×1,200), (i) glass beads (×250)

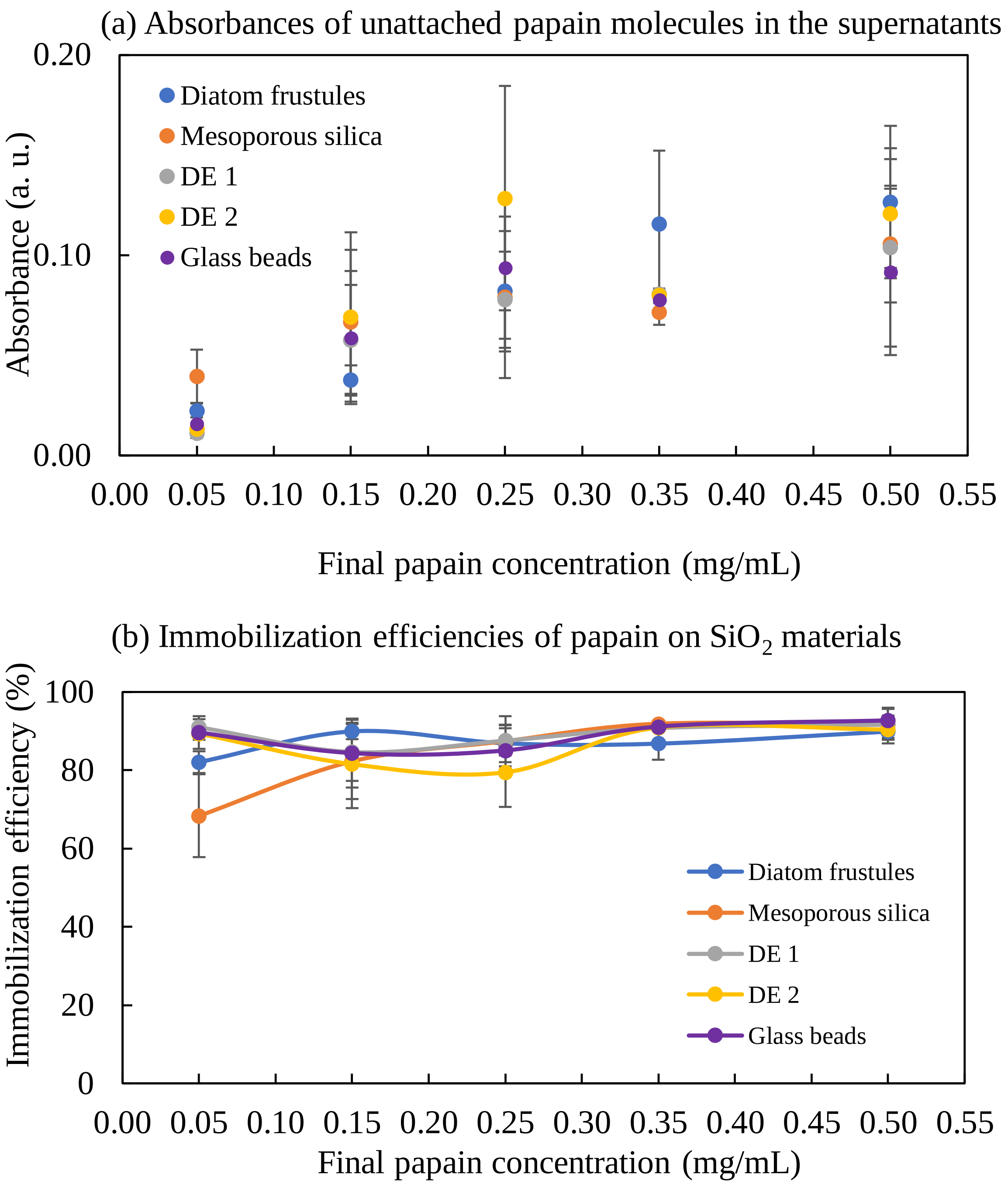

Figure S4

Attachment of papain molecules to functionalized SiO_2 materials.

Investigation of optimal concentration of papain added to SiO_2 surfaces. The initial concentration of SiO_2 was 1 mg/mL. Excess papain refers to the supernatant of the papain-SiO_2 material suspension after 2 h of mild shaking and was collected by centrifugation. Error bars indicate standard error values.

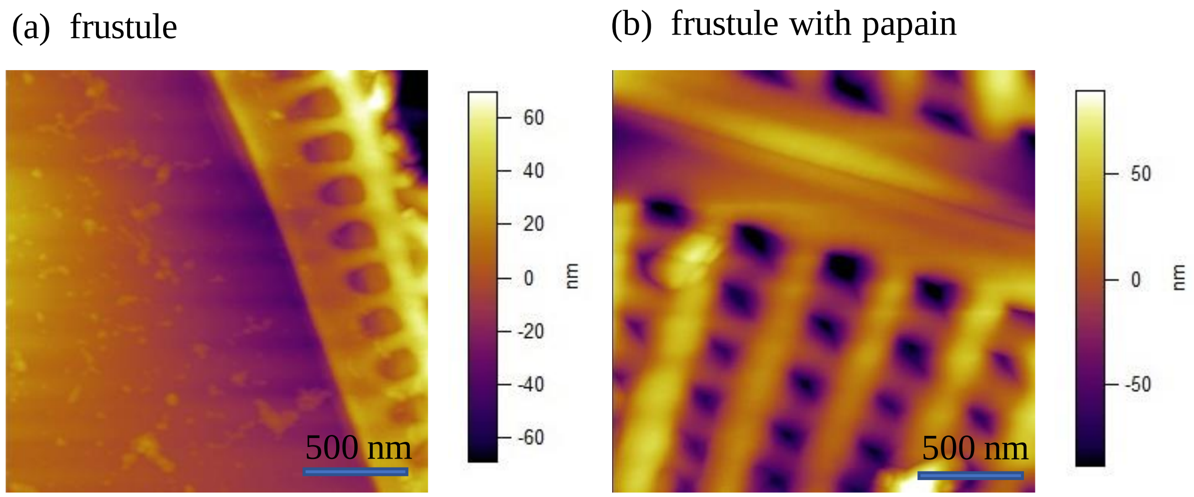

Figure S5

AFM images of (a) frustules and (b) frustules attached with papain molecules.

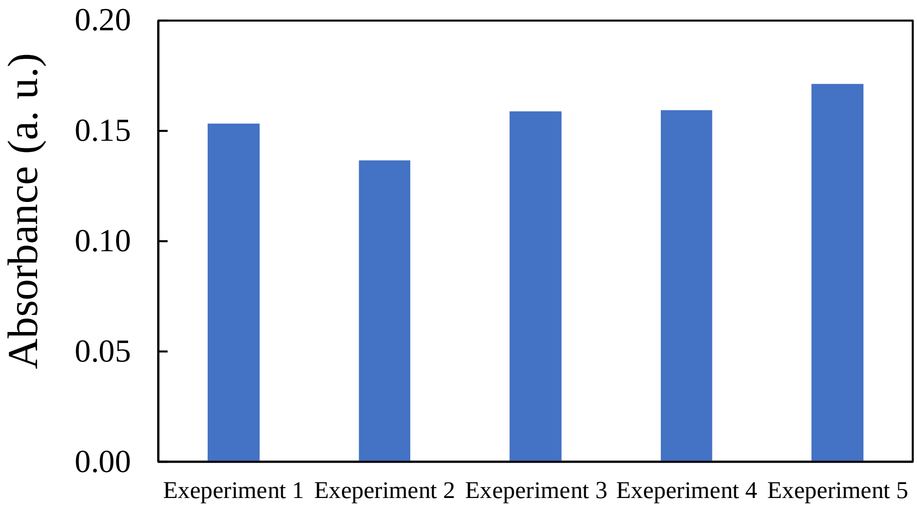

Figure S6

Absorbances showing enzyme activity of free papain.

Absorbances of pNA substrates which reacted to free papain were measured at 405 nm five times to analyze the enzyme activity of the free papain. Free papain was mixed with MES buffer (pH 7.0, 10 mM) in a microtube, and the enzyme activity was performed immediately.

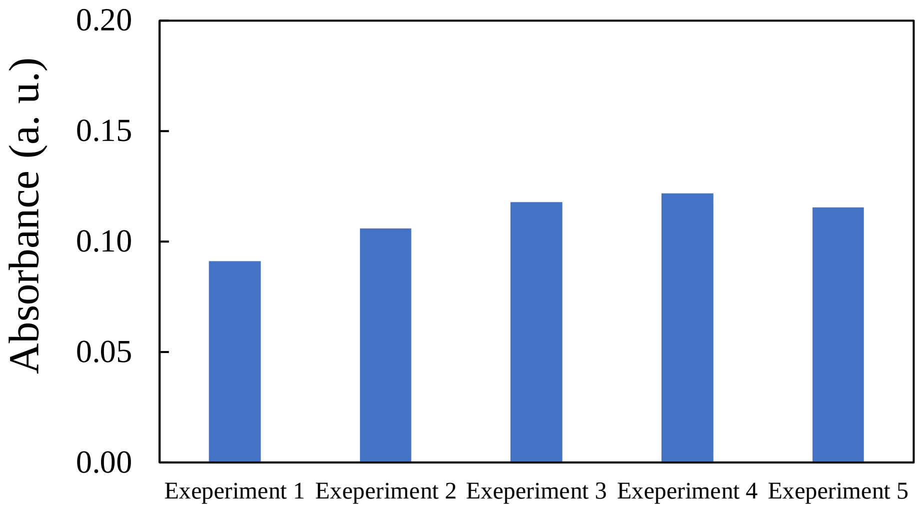

Figure S7

Absorbances showing enzyme activity of the fabricated enzyme device using frustules.

Absorbances of pNA substrates which reacted to the frustule device were measured at 405 nm five times to analyze the enzyme activity of the frustule device. The same papain was used to fabricate the enzyme device. However, papain molecules were attached onto the frustules under the MES buffer (pH 7.0, 10 mM) medium and the suspension was mixed using a rotator at room temperature for 2 h. After removing unattached papain on the frustules by centrifugation, the enzyme activity was performed.

Table S1: Absorbance of pNA substrates (measured at 405 nm) reacted with papain adsorbed on five types of SiO_2 materials and estimated enzyme activity compared to that of the frustule device at 60 °C, 200 rpm.

Enzyme devices	Room Temperature, 0 rpm		37 °C, 0 rpm		60 °C, 0 rpm		60 °C, 200 rpm	
	Absorbance (a. u)	%	Absorbance (a. u)	%	Absorbance (a. u)	%	Absorbance (a. u)	%
with diatom frustules	0.095 ± 0.073	42.8	0.114 ± 0.056	51.4	0.211 ± 0.031	95.0	0.222 ± 0.045	100
with mesoporous silica	0.087 ± 0.066	39.2	0.109 ± 0.057	49.1	0.140 ± 0.036	63.1	0.161 ± 0.067	72.5
with DE1	0.039 ± 0.033	17.6	0.053 ± 0.029	23.9	0.098 ± 0.006	44.1	0.103 ± 0.021	46.4
with DE 2	0.069 ± 0.068	31.1	0.091 ± 0.064	41.0	0.156 ± 0.037	70.3	0.161 ± 0.030	72.5
with glass beads	0.055 ± 0.052	24.8	0.055 ± 0.034	24.8	0.100 ± 0.017	45.0	0.101 ± 0.040	45.5

Table S2: Values for adsorption capacity of papain on SiO_2 materials.

Final Concentration of Papain (mg/mL)	Final Concentration of SiO_2 (μg/mL)	Adsorption Capacity of Papain on SiO_2 (mg/mg)				
		Diatom frustules	Mesoporous silica	DE 1	DE 2	Glass beads
0.05	412.5	0.10 ± 0.01	0.08 ± 0.02	0.11 ± 0.00	0.11 ± 0.00	0.11 ± 0.01
0.15	412.5	0.33 ± 0.01	0.30 ± 0.06	0.31 ± 0.05	0.30 ± 0.07	0.31 ± 0.06
0.25	412.5	0.53 ± 0.05	0.53 ± 0.07	0.53 ± 0.04	0.48 ± 0.09	0.52 ± 0.06
0.35	412.5	0.74 ± 0.06	0.78 ± 0.01	0.77 ± 0.01	0.77 ± 0.00	0.77 ± 0.00
0.5	412.5	1.09 ± 0.06	1.11 ± 0.05	1.11 ± 0.08	1.10 ± 0.05	1.12 ± 0.07

Table S3: Absorbances of pNA substrates (measured at 405 nm) reacted to the immobilized papain on the five SiO_2 materials at 200, 500, 1000 and 1500 rpm, and 60 °C

Enzyme devices	200 rpm		500 rpm		1000 rpm		1500 rpm	
	Absorbance (a. u.)	Enzyme activity (%)	Absorbance (a. u.)	Enzyme activity (%)	Absorbance (a. u.)	Enzyme activity (%)	Absorbance (a. u.)	Enzyme activity (%)
with diatom frustules	0.195 ± 0.074	100	0.150 ± 0.052	82.5	0.176 ± 0.059	94.8	0.192 ± 0.095	100.8
with mesoporous silica	0.119 ± 0.055	63.0	0.141 ± 0.073	72.6	0.084 ± 0.058	39.8	0.061 ± 0.023	34.6
with DE 1	0.063 ± 0.026	33.2	0.051 ± 0.023	26.0	0.053 ± 0.031	25.8	0.063 ± 0.039	30.7
with DE 2	0.089 ± 0.068	47.3	0.059 ± 0.018	33.3	0.119 ± 0.075	63.7	0.109 ± 0.070	55.3
with glass beads	0.045 ± 0.014	24.0	0.037 ± 0.015	19.5	0.044 ± 0.024	21.4	0.052 ± 0.030	25.1

Table S4: Reusability of the enzyme devices using frustules.

Number of uses	Absorbance (a. u.)	%
1	0.314 ± 0.028	100
2	0.290 ± 0.028	92.5
3	0.280 ± 0.034	89.7
4	0.254 ± 0.044	80.9
5	0.256 ± 0.034	81.5
6	0.206 ± 0.031	65.9
7	0.117 ± 0.024	37.2
